# Management of Impacted Maxillary Canine with Immediate Implant and Sticky Bone Auto Tooth Graft

**DOI:** 10.1155/2023/2761700

**Published:** 2023-06-23

**Authors:** Wajeha Albatal, Tarek Qasem, Jihad Jaalouk, Ghaidaa Khaddour, Yasser Alsayed Tolibah

**Affiliations:** ^1^Department of Periodontology, Faculty of Dentistry, Damascus University, Damascus P.O. Box 3062, Syria; ^2^Department of Pediatric Dentistry, Faculty of Dentistry, Damascus University, Damascus P.O. Box 3062, Syria

## Abstract

The management of the upper impacted canines includes a range of options, including orthodontic options in their various forms, up to extraction and replacing the tooth with a dental implant. Auto tooth graft (ATG) has achieved good clinical efficacy and was recently used as a grafting material for its bone induction and conduction properties. The use of platelet-rich fibrin (PRF) is highly effective in regenerative dentistry, and its use with bone grafts has improved tissue healing. This case report shows for the first time managing impacted canine with extraction and converting it into ATG and mixing it with injectable PRF to obtain sticky bone ATG and insertion of an immediate implant in a female patient who complains about a missing upper left canine. The results show the good bone formation and satisfactory clinical aspects.

## 1. Introduction

Impaction of maxillary canines is a common case [1], with a prevalence ranging from 1% to 3% [2]. Tooth impaction is defined as a condition in which the tooth remains embedded in the oral mucosa or intraosseous structures after its natural eruption appointment [3]. The maxillary canines are the second most frequently impacted teeth (2%) after third molars and most often impacted palatally (2 : 1) [4]. Impactions of the upper canines occur more often in females than in males [4]. The long pathway that the canines take as they emerge into the maxillary skeleton makes them vulnerable to disruption during their natural emergence time, at the age of 11–12 years [5, 6]. Impacted teeth have many consequences, for example, they cause migration of the neighboring teeth and loss of arch length. Moreover, impacted canines may cause root resorption of the nearby lateral incisors and they may increase a patient's risk of developing a cystic lesion and infection [2]. There are different diagnostic methods and many treatment ways for impacted upper canines, ranging from traditional radiography to cone-beam computed tomography (CBCT). Different methods were suggested to manage this condition: interceptive approaches, space recreation, orthodontic mechanical eruption, and extraction, where each method has its indications and contraindications based on several criteria within a specific age range [7]. Extraction is indicated when no other treatment plan is feasible, deeply displaced teeth, severely dilacerated, closed apex, and ankylotic teeth, followed by either space closure or implant restoration [7]. Immediate implant placement in anterior teeth had been introduced in the late 1970s by Schulte and Heimke [8]. It can avoid buccal bone resorption and reduce the period of the treatment plan [8]. It allows the surgeon to idealize the position of the implant appropriately with the better rehabilitation of normal contour to the facial aspect of the final restoration [9]. Chairside preparation of autogenous fresh demineralized teeth after extraction can be a useful alternative to the use of autogenous bone or other graft materials during implantation [10]. Auto tooth graft (ATG) was first developed in Korea in 2008 and showed good clinical and histological results [11]. Dentine has been used as a bone substitute, as it is similar to autogenous bone and it has a mineral content higher than any material derived from bone [12]. Because of that, it is considered an ideal bioactive material for hard tissue regeneration [12]. Several techniques for platelet concentrates have been introduced in the surgical field for the prevention of hemorrhage and acceleration of tissue regeneration [13]. Platelet-rich fibrin (PRF) is a second-generation platelet concentrate that was introduced by Choukroun et al. [14]. It contains various growth factors that are believed to contribute to periodontal regeneration including the platelet-derived growth factor (PDGF), insulin-like growth factor (IGF), transforming growth factor (TGF), epidermal growth factor (EGF), fibroblast growth factor (FGF), and bone morphogenetic protein (BMP) [15]. Injectable platelet-rich fibrin (i-PRF) is a liquid formulation of PRF [16], where it can stimulate tissue regeneration [17].

This case report highlights a unique case that includes replacing the impacted canine with a sticky bone ATG and immediate implant with exact details of the work performed.

## 2. Case Presentation

**Figure 1:**
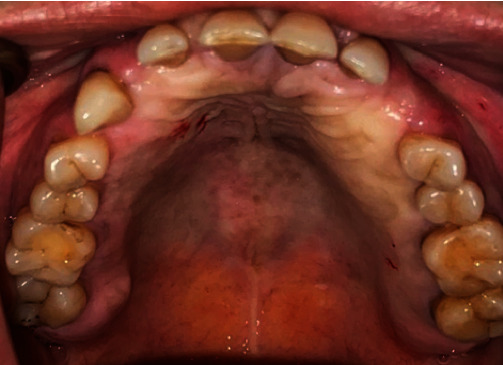
Missing upper left.

**Figure 2:**
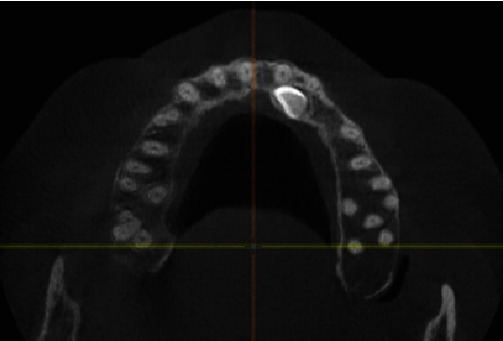
CBCT examination.

A 43-year-old female patient was referred due to a missing upper left canine (Figure 1). The patient refused the orthodontic treatment. The medical history was taken, and the necessary clinical and radiographs were taken. Routine blood tests were performed. The results came clear, where the patient was healthy and fitted for the treatment. CBCT (Vatech, Gyeonggi-do, Korea) revealed the presence of an impacted left upper canine palatally (Figure 2).

### 2.1. First Stage

**Figure 3:**
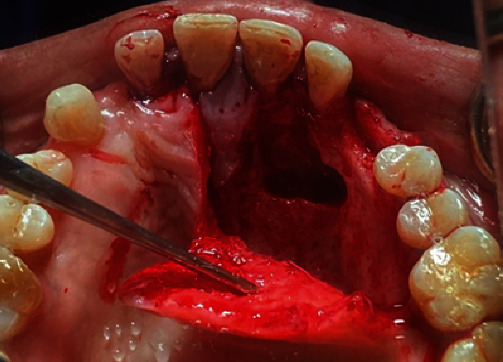
The extracted canine.

Written informed consent has been obtained. Local anesthetic was injected buccally and palatially (2% Lidocaine and 1 : 100,000 adrenaline) (Huons Lidocaine HCL, Seoul, Korea). A full-thickness palatal mucoperiosteal flap was designed, from the mesial of the left first molar to the distal of the right lateral incisor. After reflecting flap, a palatal approach osteotomy was performed by a rounded carbide bur (Dentsply Sirona Inc, Charlotte, North Carolina, USA) under copious irrigation by saline to expose the crown of the upper cuspid. After luxation of the canine with a periotome elevator (Dentsply, Tusla, OK, USA), it was separated into two pieces by the rounded carbide bur, then it was extracted atraumatically (Figure 3). The apical and ridge crest bone was intact, but the remaining palatal bone after canine extraction was defective. Meanwhile, the canine (crown and root) was turned into an ATG. The tooth was cleaned of any soft tissue by a surgical blade. The dental pulp was removed from the crown and root of the canine using the round carbide bur. The crown of the tooth was crushed into particles with a hammer in a Bone Crusher (VacuaSonic®, DecalSi-PDM®, and DecalSi-DM®; Cosmobiomedicare, Seoul, Korea). The 3 mm of end of the dental root was removed. Several small holes were made in the root at intervals of 3 mm after splitting it into two parts by vertical cut. The entire chairside preparation process, including demineralization, sterilization, and washing, was completed within 30 minutes (powder type) and 2 hours (block type) according to the manufacturer's instructions using an ultrasonic device and reagents (VacuaSonic®, DecalSi-PDM®, and DecalSi-DM®; Cosmobiomedicare, Seoul, Korea).

To make the sticky bone and the PRF membranes, venous blood was drawn from the patient's arm at a volume of 30–40 cc and distributed into three tubes with a black cap and one tube with an orange cap with a capacity of 10 ccs each [13]. The blood in the black cap tubes was used to make PRF membranes, where it was centrifuged at 3000 rpm for 10 minutes [18], while the blood in the orange cap tube was used to make the i-PRF when centrifuged at a speed 700 rpm for 3 minutes [19] in the centrifuge (Laboratory centrifuge EBA 200 series Andreas Hettich GmbH & Co.KG, 78532 Tuttlingen, Germany). i-PRF was mixed with ATG particles to make the sticky bone ATG.

**Figure 4:**
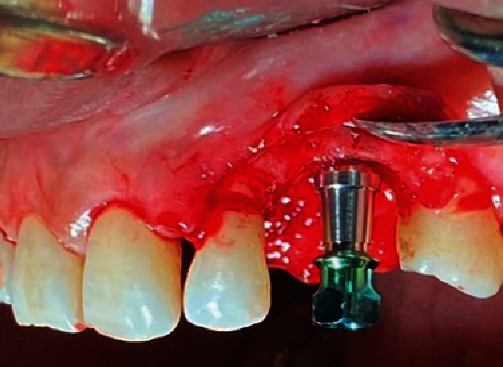
Implant Direct Legacy 2™ after insertion.

After completing the preparation of the ATG and PRF membranes, the procedure was directed to prepare the implant osteotomy after elevation of a buccal flap in the coronal region of the canine site under the abundant irrigation of saline. An implant (Implant Direct Legacy 2™, Thousand Oaks, California, USA) measuring 4.2 mm in diameter and 10 mm in length was inserted according to the manufacturer's surgical instructions, with an increasing torque up to 32 NCM [1], with good primary stability (Figure 4).

**Figure 5:**
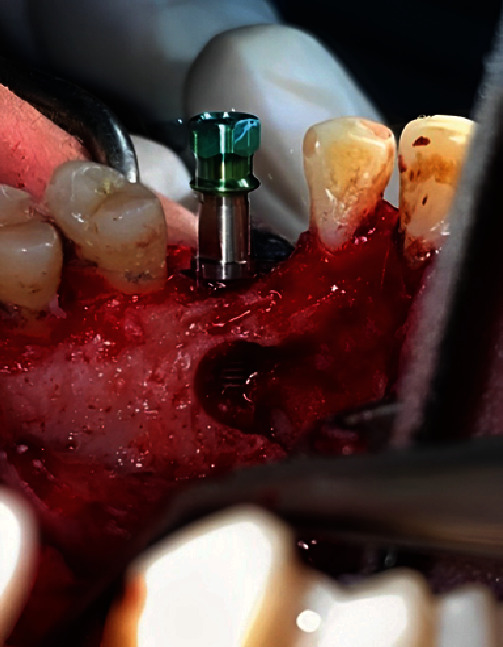
Palatally bone deficiency.

**Figure 6:**
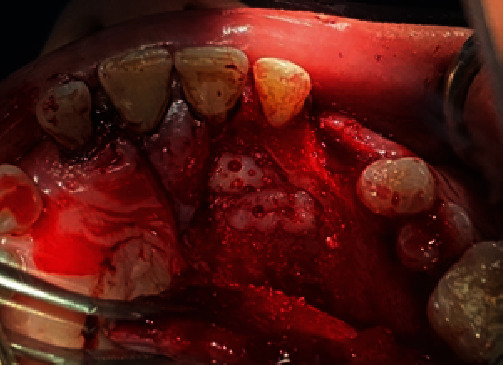
Sticky bone ATG and blocks of ATG on implant.

**Figure 7:**
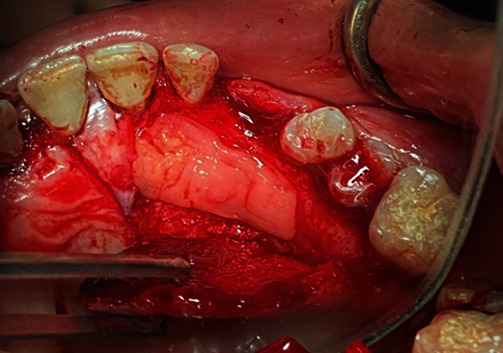
PRF membranes on grafts.

**Figure 8:**
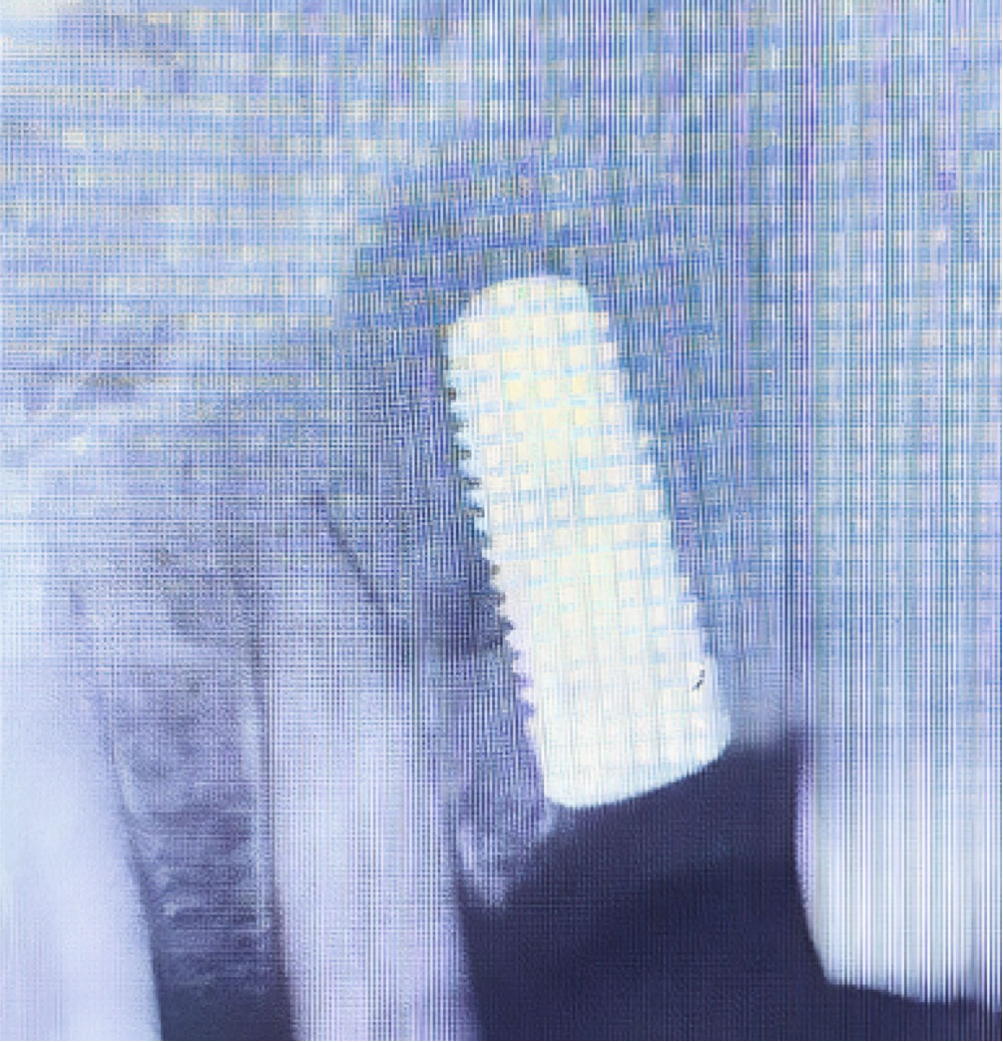
Periapical radiograph.

A bone deficiency remained palatally, where part of the circumference of the implant was left without bone coverage, except for the apical and coronal areas (Figure 5), so the implant screw threads and the bone defect around the implant were covered by sticky bone ATG and blocks of ATG, followed by PRF membranes (Figures 6 and 7). Nylon 4-0 suturing (Jinhuan Medical Products Co. Ltd., Shanghai, China) was used to close the flap. A periapical radiograph was taken (Figure 8). Amoxicillin with clavulanic acid was prescribed (1000 mg, tab, 2 times a day for 7 days), Ibuprofen (400 mg, tab, 3 times a day after food for 4 days), and 0.2% chlorhexidine rinses.

### 2.2. Second Stage

The sutures were removed 10 days after the operation, and the postoperative healing course was normal. It was decided to make the final restoration after 3 months.

### 2.3. Third Stage

**Figure 9:**
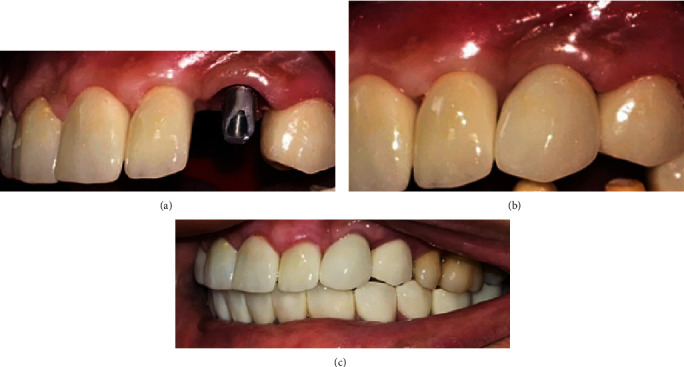
Final prosthesis. (a) An abutment on implant. (b) Final prosthesis on the canine after cementation. (c) The final view of zirconia crowns after 12 months.

After 3 months, final prosthesis was cemented. A healing abutment (Implant Direct 8735-13) was placed for 2 weeks. Impression was made with an open-tray impression by silicone material (Zhermack Zetaplus Silicone, Badia Polesine, RO, Italy). Zirconia crown was made for the canine. The patient required a mouth rehabilitation so she had zirconia crowns on all her teeth (Figure 9).

**Figure 10:**
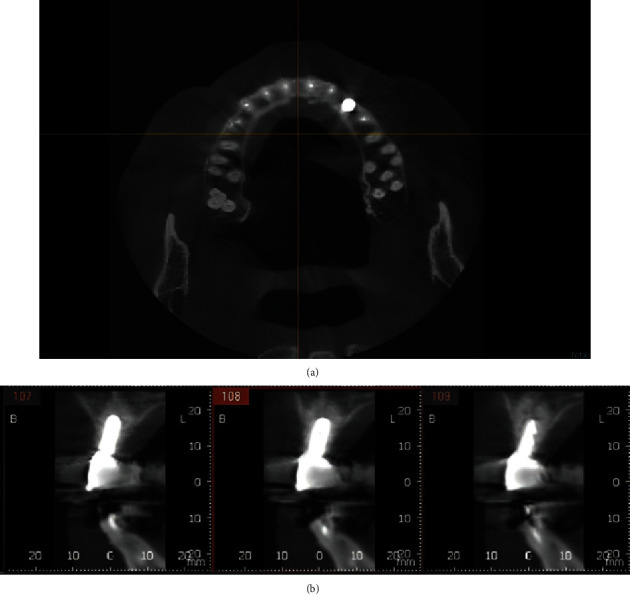
CBCT after 12 months. (a) Occlusal view. (b) Sagittal views.

CBCT after 12 months (Figure 10) showed predictable bone formation on the palatal of the implant and good osseointegration.

## 3. Discussion

The patient had impacted canine with a closed apex and was close to the incisor roots (as shown on the CBCT image), except that she did not want to perform unpredictable orthodontic treatment due to her relatively advanced age. All of these factors must be considered when managing the impacted canine [7, 20, 21].

The extraction of the impacted canine is one of the treatment options that can be resorted to when several conditions are met, where it can be extracted followed by closing the space or replacing it with a dental implant or a fixed prosthodontic [7]. Dental implant techniques and systems are becoming available today, making them more predictable, with the decreased willingness of patients to undergo orthodontics treatment [2].

According to the search of the PubMed database, this is the first use of ATG sticky bone to graft the defect left after impacted canine removal that is prepared in a special ultrasonic device (VacuaSonic®, DecalSi-PDM®, and DecalSi-DM®; Cosmobiomedicare, Seoul, Korea).

This report presents a good prognosis case of extraction of an impacted canine and replacement it with a dental implant after converting it into sticky bone ATG to graft the remaining palatal defect after implant placement, where the choice of an extraction, implantation, and grafting in this patient was similar to what other researchers did, such as Cardaropoli et al. in similar situations [1].

One of the advantages of this technique is to reduce the number of operations performed while reducing procedure time, but its disadvantages are the possibility of failure of graft then the implant [22]. There is a variety of graft materials, such as allogenic, xenogenic, and alloplastic graft materials, that have been used as alternatives. Meanwhile, autogenous bone is still considered the gold standard, but the infection of the donor site is still a problem [23]. Recently, teeth attracted attention as a material for alveolar bone regeneration, where the chemical composition of dentin closely resembles that of bone [10, 24]. Kim demonstrated that ATG could be a good material for grafting and suggested recycling a tooth into a bone graft [10]. It has osteoinductive and osteoconductive potential, as it contains mainly type-I collagen with growth factors such as bone morphogenetic proteins 2 and FGFs [11]. ATG can also give a greater amount of bone regeneration compared to xenograft [25]. In addition, the steps for preparing ATG are relatively easy, as they were following the instructions of the company that produced the tools for its preparation, and the same preparation steps were folded in the study of Kim [10]. Moreover, the study of Wu et al. and Shah et al. used autogenous tooth graft versus beta-tricalcium phosphate (*β*-TCP) alloplastic in alveolar ridge preservation, and they found that ATG as compared to *β*-TCP provided superior results, based on this, they concluded that ATG material could serve as a better alternative to conventional bone graft materials [26, 19]. Hara et al. used bone transport and bone graft using auto-tooth bone for alveolar cleft repair, and they found that for small alveolar cleft repairs, auto-tooth bone grafting by itself might be used [27]. Del Canto-Díaz et al. used autologous tooth-derived graft material in the post-extraction dental socket, and they conducted that autologous dentine might be considered a promising material for use in socket preservation techniques [12]. Wu et al. evaluated immediate implant placement in anterior teeth with grafting material of autogenous tooth bone versus xenogenic bone, and they found that the bone volume change in the facial part of the implant after immediate placement was almost the same between the two groups and provided clinical evidence that the autogenous tooth bone made from a compromised tooth could be an acceptable bone graft material [26]. Pang et al. used an autogenous demineralized dentin matrix from the extracted tooth for the augmentation of alveolar bone defect in comparison with an organic bovine bone, and they found that the two materials were effective in the augmentation of vertical dimensions so the autogenous tooth graft material was a viable option for alveolar bone augmentation following dental extraction [28]. For the first time, a sticky bone has been created by mixing ATG with i-PRF. Sticky bone ATG prepared from the canine, which is used for grafting the defect area and covering it with the PRF membranes, accelerates the regeneration of bone and soft tissues, where the sticky bone has several advantages, including moldable, which can be adapted to the desired shape and ensures good stability for graft. Growth factors present in the PRF membranes placed over the sticky bone, such as TGF, PDGF, and vascular endothelial growth factor, accelerate the bone formation and improve tissue healing [13]. Karayürek et al. evaluated combining PRF with autogenous graft, xenograft, and *β*-TCP in a rabbit model and they found that autogenous graft combined with PRF yielded superior results but the combination of *β*-TCP–PRF had no effect compared to the only-*β*-TCP [18]. Kulkarni et al. used PRF as a grafting material in periapical surgery and they found excellent bone fill in the periapical defects grafted solely with PRF [29]. Moreover, Shah et al. evaluated the biological activation of bone grafts using i-PRF and they noted that the discovery of injectable PRF might lead to the discovery of an injectable and superior alternative to PRF and Platelet-Rich Plasma for all kinds of medical and dental applications [19].

The occlusion in terms of occlusal form and scheme design that applies to implants is different from the occlusion that applies to natural teeth [30]. On implants, it has to be designed to reduce the occlusal force during axial and non-axial loading conditions, as well as the stress around the implant and supporting bone structures [30]. The modified occlusion concept on implants includes a rounded and diminished cusp tip, narrow occlusal table, smooth grooves and fossa, slight occlusal contact, and shallow or flat occlusal anatomy [30] so that this was in our mind when designing the final prosthesis in this report. Space closure after the impacted canine removal can be done, either protracting the premolars in the canine position and reshaping them [7] or fixed prosthetic between the lateral incisor and first premolar but the patient rejected this solution. The device for preparing the graft may be not found in all clinics, and the length of the procedure may be the limitation of this technique. Recently, there are some materials and therapies that can have a significant influence on the oral environment like ozonized water, laser photodynamic with a Light Emitting Diode (LED) or a laser, and an antimicrobial gel containing postbiotics, lactoferrin, and *Aloe barbadensis* leaf juice powder [31, 32, 33].

These therapies can modify clinical and microbiological parameters in periodontal patients [31, 32, 33]. Butera et al. used ozonized water, as it had many properties such as immunomodulant, anti-hypoxic, anti-inflammatory, and regenerative ones in peri-implant mucositis sites, and they found that the application of ozonized water on peri-implant mucositis sites had significantly reduced all the clinical indexes, with a progressive improvement from baseline to the subsequent assessments at 1 month and 2 months, except for the plaque index, which only improved at 1 month without a further subsequent significant reduction [31]. Koochaki et al. evaluated the effects of antimicrobial photodynamic therapy (PDT) with an LED and a laser on the proliferation of human gingival fibroblasts on the root surface, as it had main advantages over the conventional antimicrobial therapies include the immediate onset of action, elimination of resistant microorganisms, and secreted virulence factors, and they found that PDT with toluidine blue (TBO) activated by an LED and a laser and citric acid treatment as an adjunct to periodontal therapy could enhance the proliferation of human gingival fibroblasts on dentin blocks [32]. Butera et al. compared an antimicrobial gel with postbiotics, lactoferrin, and *A. barbadensis* leaf juice powder in compared to conventional chlorhexidine gel in periodontal patients as a home oral care material, and they found that postbiotics were supposed to be a natural alternative for traditional chemical substances like chlorhexidine [33]. We could use these advanced therapies with our technique as they could have a positive effect on the results but because of their limited availability, we did not use them, and this was one of the limitations of this report. Further studies about these therapies with bone regeneration procedures are needed.

## 4. Conclusion

The extraction of impacted canines in some indicated cases, then replacing them with dental implants with grafting by sticky bone ATG, may be a good option. It is a unique treatment technique for impacted canines in an adult who have limitations for orthodontic treatment. CBCT is very important in cases like this to evaluate the success of this treatment. More studies are required to evaluate the results of this technique over time.

## Data Availability

Data supporting this research article are available from the corresponding author or first author on reasonable request.
